# Supplementary material for: WordCommentsAnalyzer: A windows software tool for qualitative research
Source: F1000Res. 2018 Sep 4;7:536. Originally published 2018 May 3. [Version 2] doi: 10.12688/f1000research.14819.2 (PMC6137411; doi:10.12688/f1000research.14819.2)
Supplement: Supplementary file 3 [file f1000research-7-17614-s0002.tgz › b2a79496-4ada-47c4-9682-a99ce867836d.zip!/Eight Iranian Health Journals/Journal of Education and Community Health_13.docx]

© Journal of Education and Community Health. This work is licensed under CC BY-NC 4.0 (https://creativecommons.org/licenses/by-nc/4.0/).

Volume & Issue: دوره 1 - شماره 4

Title: تأثیر شیوه آموزشی مبتنی بر بحث گروهی بر ارتقاء رفتارهای بهداشتی دوران عادت ماهیانه دختران نوجوان ساکن مراکز شبانه‌روزی بهزیستی شهر تهران

http://jech.umsha.ac.ir/article-1-63-fa.html

سابقه و هدف: مشکلات مربوط به عادت ماهیانه یکی از علل عمده ناخوشی در زنان است. بحث گروهی یک روش پژوهشی است که گروه کوچکی از مشارکت‌کنندگان به منظور گردآوری اطلاعات به بحث درباره یک موضوع خاص می‌پردازند. این پژوهش با هدف تعیین تأثیر آموزش از طریق بحث گروهی بر رفتارهای بهداشتی دوران عادت ماهیانه دختران نوجوان انجام گرفت.  مواد و روش ها: پژوهش حاضر یک مطالعه مداخله‌ای نیمه تجربی (یک گروهی) می‌باشد، که بر روی دختران 19-12 سال ساکن مراکز شبانه‌روزی بهزیستی شهر تهران انجام شد. در این مطالعه کلیه افراد واجد شرایط (61 نفر) وارد مطالعه شدند. ابزار گردآوری اطلاعات پرسشنامه محقق ساخته شامل دو قسمت خصوصیات دموگرافیک و سؤالات عملکرد در خصوص بهداشت بلوغ جسمی (دوران عادت ماهیانه) بود که قبل و یک ماه پس از مداخله تکمیل گردید. مداخله آموزشی در طی 3 ماه انجام گرفت. داده‌ها با استفاده از آزمون‌های آماری کولموگروف-اسمیرنوف و آزمون تی‌زوجی از طریق نرم‌افزار SPSS-16 تحلیل شدند.  یافته‌ها: پس از مداخله آموزشی در مقایسه با قبل از آن میانگین نمره عملکرد از 4.43±12.11 به 2.97±16.50 افزایش یافت که این میزان تغییر از لحاظ آماری معنی‌دار بود (P<0.001).  نتیجه‌گیری: مداخله آموزشی از طریق بحث گروهی تأثیر مثبتی در بهبود عملکرد افراد مورد مطالعه در خصوص بهداشت بلوغ جسمی داشت، لذا استفاده از این روش در آموزش مسائل مربوط به دوران بلوغ دختران نوجوان پیشنهاد می‌شود.

Title: عوامل پیش‌بینی‌کننده رفتارهای تغذیه‌ای بر اساس تئوری شناختی اجتماعی در زنان باردار مراجعه‌کننده به مراکز بهداشتی و درمانی شهر تبریز

http://jech.umsha.ac.ir/article-1-59-fa.html

سابقه و هدف: تغذیه مادران در طول بارداری عامل کلیدی در تضمین سلامت مادر و کودک است. لذا مطالعه حاضر با هدف بررسی عوامل پیش‌بینی‌کننده رفتارهای تغذیه‌ای در بین زنان باردار شهر تبریز بر اساس تئوری شناختی اجتماعی انجام‌ شده است. مواد و روش‌ها: این مطالعه تحلیلی در سال1393 با مشارکت 380 زن باردار مراجعه‌کننده به 10مرکز بهداشتی درمانی شهر تبریز انجام شد که به روش تصادفی چندمرحله‌ای انتخاب و وارد مطالعه شدند. معیار ورود زنان باردار به این مطالعه قرار داشتن در محدوده سنی 15 تا 45 سال و نداشتن هرگونه بیماری جسمی، روحی حاد و مزمن بود. ابزار گردآوری داده‌ها شامل پرسشنامه استاندارد رفتارهای تغذیه‌ای و پرسشنامه محقق ساخته سازه های تئوری شناختی اجتماعی (آگاهی، انتظارات پیامد، ارزش‌های پیامد، خودکارآمدی، حمایت اجتماعی و خودتنظیمی) بود که به روش مصاحبه و خودگزارش‌دهی تکمیل گردید.   یافته‌ها: میانگین سنی شرکت‌کنندگان 27 سال بود. از میان سازه های مختلف تئوری شناختی اجتماعی، انتظارات پیامد (P<0.039)، ارزش های پیامد (P<0.046)، آگاهی (P<0.043) و خودتنظیمی (P<0.001) دارای سهم معنی‌داری در تبیین واریانس رفتار تغذیه ای مناسب در بین مادران مورد مطالعه بودند. این متغیرها در مجموع 16 درصد از واریانس رفتارهای تغذیه‌ای را پیش‌بینی گردید. در 54.4% افراد مصرف نان و غلات، 73.2% دریافت گوشت و حبوبات ، 55.3% مصرف میوه‌جات و در 70.5% مصرف سبزیجات کمتر از میزان توصیه شده بود.   نتیجه گیری: نتایج نشان داد، آگاهی، انتظارات پیامد، ارزش های پیامد و خودتنظیمی می‌توانند جهت طراحی مداخلات آموزشی برای دستیابی به رفتارهای تغذیه ای سالم در زنان باردار مؤثر باشد.

Title: انطباق برخی سازه های مدل های رفتاری با دلایل اقدام به ترک اعتیاد : یک مطالعه کیفی

http://jech.umsha.ac.ir/article-1-79-fa.html

سابقه و هدف: جمعیت جهان بیش از هفت میلیارد نفر می‌باشد که از این میان 230 میلیون نفر آن دچار سوءمصرف مواد هستند. برنامه‌های پیشگیری و درمان سوءمصرف مواد، طی دو دهه اخیر مورد توجه قرار گرفته است. یک موضوع بسیار اساسی در ترک اعتیاد، علت مراجعه و اقدام به آن می‌باشد. شناخت دلایل اولیه اقدام به ترک در نتیجه‌گیری از درمان نیز بسیار مهم می باشند. هدف این مطالعه شناسایی این عوامل و تطبیق آنها با برخی از سازه‌های مدل‌های رفتاری است. مواد و روش‌ها: این مطالعه با رویکرد کیفی و به روش تحلیل محتوا انجام شد. مشارکت‌کنندگان 21 نفر بیمار، پزشک و کارشناس بودند که از چند کلینیک ترک اعتیاد شهر بجنورد در سال 93 انتخاب شدند. نمونه‌گیری مبتنی بر هدف و تا اشباع داده‌ها ادامه داشت. برای جمع‌آوری داده‌ها از مصاحبه نیمه ساختارمند و یادداشت‌برداری استفاده شد، مصاحبه‌ها ضبط و پیاده‌سازی شدند.  یافته‌ها: با تحلیل داده‌ها، سی ‌و سه طبقه فرعی و نه طبقه اصلی به دست آمد که شامل: مشکلات اقتصادی، دغدغه‌های ناشی از خود مواد، مشکلات و عوارض فردی، مشکلات و عوارض خانوادگی، مشکلات و عوارض اجتماعی، انتظارت خانواده، توجه به موقعیت اجتماعی، باور نسبت به اعتیاد و ارزش‌گذاری به رفتار ترک استخراج شد. سپس چهار درون‌مایه تهدید درک‌شده، موانع درک‌شده در تداوم مصرف، نگرش به رفتار و هنجارهای انتزاعی تشخیص داده شد.  نتیجه‌گیری: دلایل مرتبط با اقدام به ترک اعتیاد با سازه‌های مدل‌ها و نظریه‌های مختلف آموزش بهداشت از جمله برخی سازه‌های مدل اعتقاد بهداشتی و نظریه رفتار برنامه‌ریزی ‌شده تطبیق دارند.

Title: ارزشیابی برنامه آموزش پیشگیری از آسیب‌های ناشی از حوادث مدرسه‌ای در دانش آموزان ناشنوا و نیمه شنوای دبیرستانی بر اساس مدل اعتقاد بهداشتی

http://jech.umsha.ac.ir/article-1-69-fa.html

سابقه و هدف: دانش آموزان یکی از گروه‌های آسیب‌پذیر در مقابل حوادث می‌باشند. دانش آموزان ناشنوا و نیمه شنوا به علت معلولیتشان ممکن است نسبت به دانش آموزان عادی آسیب‌پذیرتر باشند. لذا هدف این مطالعه ارزشیابی برنامه آموزش پیشگیری از آسیب‌های ناشی از حوادث مدرسه‌ای در دانش آموزان ناشنوا و نیمه شنوای مقطع دبیرستان بر اساس مدل اعتقاد بهداشتی بود.  مواد و روش‌ها: مطالعه نیمه تجربی حاضر در بین کلیه دانش آموزان ناشنوا و نیمه شنوای دبیرستانی شهر همدان (دو مدرسه) در سال1392 انجام شد. دانش آموزان به روش تصادفی در دو گروه مداخله (23نفر) و کنترل (27نفر) قرار گرفتند. داده‌ها توسط پرسشنامه‌ای شامل سؤالات مربوط به متغیرهای جمعیت‌شناختی، سازه‌های مدل اعتقاد بهداشتی، آگاهی و رفتارهای پیشگیری‌کننده به‌صورت خودگزارش‌دهی، قبل و دو ماه بعد از مداخله آموزشی در دو گروه تکمیل گردید. مداخله آموزشی شامل ارائه یک کتابچه طراحی‌شده و برگزاری 5 جلسه آموزشی بود. برای تحلیل داده‌ها از نرم‌افزار SPSS-16 و آزمون‌های تی‌زوجی، فیشر، تی‌مستقل و آزمون کای‌دو استفاده شد. یافته‌ها: یافته‌ها نشان داد بعد از مداخله آموزشی افزایش میانگین نمرات آگاهی (P=0.002)، رفتارهای پیشگیری‌کننده (P=0.001) و سازه‌های مدل اعتقاد بهداشتی شامل شدت درک‌شده (P=0.001)، منافع درک‌شده (P=0.001)، خودکارآمدی (P=0.001) و راهنما برای عمل (P=0.001) در گروه مداخله در مقایسه با گروه کنترل معنی‌دار بود.  نتیجه‌گیری: نتایج مطالعه نشان داد که مداخله آموزشی بر مبنای مدل اعتقاد بهداشتی می‌تواند سبب ارتقاء رفتارهای پیشگیری‌کننده از آسیب‌های ناشی از حوادث مدرسه‌ای در دانش آموزان ناشنوا و نیمه شنوا گردد.

Title: آگاهی، نگرش و عملکرد مادران در خصوص نشانه های خطر عفونت های تنفسی و اسهال در کودکان کمتر از پنج سال شهر اراک در سال 1393

http://jech.umsha.ac.ir/article-1-73-fa.html

سابقه و هدف: بیشتر بیماری‌های کودکان دارای علائم و نشانه‌هایی هستند که برخی از این علائم، نشانه‌ی خطر می‌باشند. دانش مادران نسبت به علائم و نشانه‌های بیماری‌ها بخصوص بیماری‌های تنفسی و گوارشی، در کاهش میزان مرگ و میر کودکان کمتر از پنج سال کمک‌کننده است. لذا پژوهش حاضر باهدف تعیین آگاهی، نگرش و عملکرد مادران در مورد نشانه‌های خطر در کودکان کمتر از پنج سال انجام شد.  مواد و روش‌ها: این مطالعه توصیفی-تحلیلی بر روی 350 نفر از مادران دارای کودک کمتر از پنج سال مراجعه‌کننده به مراکز بهداشتی درمانی شهر اراک که به صورت تصادفی ساده انتخاب شدند انجام پذیرفت. ابزار گردآوری اطلاعات، پرسشنامه محقق ساخته چندقسمتی شامل مشخصات جمعیت‌شناختی، آگاهی، نگرش و عملکرد بود که روایی و پایایی آن قبل از مطالعه بررسی شد. داده‌ها از طریق مصاحبه جمع‌آوری و با استفاده از ویرایش 19 نرم‌افزار SPSS و آزمون‌های همبستگی پیرسون، تی‌مستقل و آنالیز واریانس یک‌طرفه تجزیه‌ و تحلیل شدند.  یافته‌ها: میانگین و انحراف معیار نمره آگاهی مادران در مورد نشانه‌های خطر کودکان 13.7±60.17، نگرش 5.29±77.45 و عملکرد در زمینهٔ عفونت‌های تنفسی 41.5±49.5، اسهال 41.42±49.25 و تب و تشنج 42.91±52.1 بود. از نظر عملکرد بین مادران شاغل و خانه‌دار تفاوت معنی‌داری وجود داشت. عملکرد مادران در وضعیت‌های اقتصادی و سطح تحصیلات مختلف تفاوت معنی‌داری داشت. بین نگرش با آگاهی و عملکرد رابطه مثبت معنی‌داری مشاهده گردید (P<0.001).  نتیجه‌گیری: یافته‌های این مطالعه می‌تواند برای طراحی و اجرای برنامه‌های آموزشی با هدف ارتقای آگاهی، نگرش و عملکرد مادران در مورد نشانه‌های خطر کودکان مفید باشد.

Title: بررسی رابطه همسرآزاری و سلامت روانی در زنان دچار خشونت خانگی ارجاع شده به پزشکی قانونی شهر شیراز

http://jech.umsha.ac.ir/article-1-82-fa.html

سابقه و هدف: بی‌توجهی به سلامت زنان و اعمال خشونت خانگی علیه آنان می‌تواند سبب پیدایش انواع بیماری‌های روانی و حتی جسمانی گردد که این امر تهدیدی علیه سلامت اعضای خانواده است. اختلال در روند زندگی زنان و مادران نتایج سویی را نه تنها برای خانواده، بلکه بر کل سلامت جامعه خواهد گذاشت. در این راستا پژوهش حاضر با هدف بررسی رابطه همسر آزاری و سلامت روانی زنان دچار خشونت خانگی مراجعه‌کننده به پزشکی قانونی شهر شیراز انجام شد.  مواد و روش‌ها: این مطالعه از نوع مطالعه توصیفی– تحلیلی از نوع مقطعی می‌باشد. حجم نمونه معادل 197 نفر از زنان دچار همسر آزاری ارجاع شده به پزشکی قانونی استان فارس، طی سال 1392 در 3 ماه به دست آمد. ابزار گردآوری داده‌ها شامل پرسشنامه جمعیت‌شناختی، پرسشنامه سلامت عمومی (28-GHQ) و پرسشنامه استاندارد خشونت علیه زنان بود، جهت برآورد پایایی ابزارهای اندازه‌گیری، آلفای کرونباخ استفاده شد، تجزیه ‌و تحلیل داده‌ها با استفاده از ویرایش 18 نرم‌افزار SPSS انجام شد. یافته‌ها: میانگین سنی نمونه‌های پژوهش 6.27±30.42 گزارش شد. بیش از 50 درصد زنان مورد خشونت واقع شده بودند که از اختلال سلامت عمومی در تمامی ابعاد رنج می‌بردند (P<0.05). رابطه انواع همسر آزاری با هر یک از ابعاد سلامت عمومی بررسی شد، نتایج نشان داد که بین این متغیرها رابطه مثبت وجود دارد. به عبارتی خشونت از هر نوع، پیش‌بینی‌کننده بروز اختلال در سلامت عمومی فرد بود (P<0.05). در این میان بیشترین اثر را بر بعد افسردگی و اضطراب داشت.  نتیجه‌گیری: پژوهش حاضر نشانگر پیامدهای روانی همسر آزاری و خشونت علیه زنان بوده و تأییدی بر آسیب به سلامت روان زنان، بر اثر خشونت است. اختلال در سلامت روان زنان علاوه بر تحمیل هزینه‌های گزاف مراقبتی و دارویی بر جامعه، زندگی خانوادگی و تربیت فرزندان را دچار مشکل می‌کند.

Title: بررسی رفتارهای ارتقاء دهنده سلامت مادران باردار مراجعه‌کننده به واحد مراقبت‌های دوران بارداری بیمارستان امام خمینی سقز

http://jech.umsha.ac.ir/article-1-85-fa.html

سابقه و هدف: رفتارهای ارتقاء دهنده سلامت از اهمیت قابل توجهی در سلامت زنان باردار و فرزندان آنها برخوردار است. پژوهش حاضر با هدف بررسی رفتارهای ارتقاء دهنده سلامت در مادران باردار مراجعه‌کننده به کلینیک مادران باردار بیمارستان امام خمینی(ره) شهرستان سقز انجام شد.  مواد و روش‌ها: این مطالعه توصیفی-تحلیلی با مشارکت 181 مادر باردار انجام شد. نمونه ها با استفاده از روش نمونه‌گیری در دسترس انتخاب شدند. ابزار گردآوری داده‌ها مشتمل بر پرسشنامه دو بخشی بود. بخش اول مربوط به اطلاعات جمعیت‌شناختی و بخش دوم مربوط به رفتارهای ارتقاء دهنده سلامت (Health Promotion Lifestyle II) بود. داده‌ها وارد نرم‌افزار SPSS-21 شده و با استفاده از روش های آماری توصیفی (فراوانی و درصد) و استنباطی از قبیل آزمون‌های تی‌مستقل، پیرسون و آنالیز واریانس یک‌طرفه مورد تجزیه‌ و تحلیل قرار گرفت.  یافته‌ها: بر پایه یافته‌های مطالعه رفتارهای ارتقاء دهنده سلامت مادران باردار در حیطه رشد معنوی بیشترین نمره و حیطه فعالیت فیزیکی کمترین نمره را داشتند. میانگین مجموع نمرات رفتارهای ارتقاء دهنده سلامت در حد مطلوب بود. از بین متغیرهای جمعیت شناختی، تنها شغل رابطه معنی داری با رفتارهای ارتقاء دهنده سلامت داشت (P<0.032).  نتیجه‌گیری: نتایج بیانگر سطح مطلوب بودن رفتارهای ارتقاء دهنده سلامت در این گروه از زنان باردار بود. توصیه می‌شود توجه بیشتری به حیطه‌هایی مانند فعالیت فیزیکی، مدیریت تنش و مسئولیت ‌پذیری در دوران بارداری شود و مطالعات دیگر در سایر قومیت‌ها با حجم نمونه بیشتر انجام شود.

Title: ارتقاء سلامت دهان و دندان دانش آموزان 12-6 ساله: یک مرور نظام مند

http://jech.umsha.ac.ir/article-1-54-fa.html

سابقه و هدف: کودکان 12-6 ساله به علت شیوع بالای پوسیدگی دندان، تکامل دندان‌های دائمی و شکل‌گیری سبک زندگی، مهم‌ترین گروه هدف برنامه‌های بهداشت دهان و دندان هستند و مدرسه آسان‌ترین و به‌صرفه‌ترین روش دستیابی به این گروه است. این مطالعه با هدف بررسی تأثیر مداخلات مبتنی بر مدرسه بر ارتقای سلامت دهان و دندان دانش آموزان 12-6 ساله انجام شده است.  مواد و روش‌ها: جستجوی الکترونیکی در پایگاه‌های اطلاعاتیScopus, Proquest, Science Direct, Pub Med, Springer, Biomed Central, Google Scholar،Iran Medex و SID با استفاده از کلمات کلیدی فارسی و انگلیسی انجام و مداخلات آموزشی که جهت پیشگیری از بیماری‌های دهان و دندان و ارتقاء رفتارهای بهداشتی مرتبط در بین دانش آموزان اجرا و بین ژانویه 2004 تا سپتامبر 2014 چاپ شده بودند، مورد بررسی قرار گرفتند. یافته‌ها: 17 مطالعه انتخاب‌ شده به دو دسته آموزش مبتنی بر رفتارهای خودمراقبتی و ارائه خدمات پیشگیرانه و آموزش مبتنی بر رفتارهای خودمراقبتی تقسیم شدند. آن دسته از مداخلاتی که دارای ویژگی‌های زیر بودند با موفقیت بیشتری در افزایش آگاهی، رفتارهای بهداشتی، کاهش پلاک و پوسیدگی دندان همراه بودند: استمرار مداخله، درگیر کردن والدین و کارکنان مدرسه در برنامه، کاربرد مدل‌های تغییر رفتار، استفاده از ترکیب چندین روش آموزشی و به‌کارگیری تجربه عملی و خدمات پیشگیرانه.  نتیجه‌گیری: استمرار آموزش‌های اجتماع‌محور، استفاده از برنامه‌های آموزشی مدون و ترکیبی، پیگیری، استفاده از نظریه‌ها و مدل‌های تغییر رفتار و ارائه خدمات پیشگیرانه بهترین راه رسیدن به نتایج مورد انتظار است.
